# Comparison of non-invasive *Staphylococcus aureus* sampling methods on lesional skin in patients with atopic dermatitis

**DOI:** 10.1007/s10096-021-04365-5

**Published:** 2021-11-04

**Authors:** Heimo Lagler, Christine Bangert, Tamara Quint, Zoe Österreicher, Alina Nussbaumer-Pröll, Sabine Eberl, Maria Weber, Matthias Karer, Morten O. A. Sommer, Markus Zeitlinger

**Affiliations:** 1 Division of Infectious Diseases and Tropical Medicine, Department of Medicine I, Medical University of Vienna, Vienna, Austria; 2 Department of Dermatology, Medical University of Vienna, Vienna, Austria; 3 Department of Clinical Pharmacology, Medical University of Vienna, Vienna, Austria; 4 UNION Therapeutics A/S, Hellerup, Denmark; 5 DTU Biosustain, Technical University of Denmark, Kongens Lyngby, Denmark

**Keywords:** Atopic dermatitis, *Staphylococcus aureus*, Colonisation, Sampling methods, Selective media

## Abstract

There is evidence that *Staphylococcus aureus* colonisation is linked to severity of atopic dermatitis. As no gold standard for *S. aureus* sampling on atopic dermatitis skin lesions exists, this study compared three commonly used methods. In addition, effectiveness of standard skin disinfection to remove *S. aureus* colonisation from these inflamed skin lesions was investigated. In 30 atopic dermatitis patients, three different *S. aureus* sampling methods, i.e. detergent scrubbing, moist swabbing and tape stripping, were performed on naïve and disinfected skin lesions. Two different *S. aureus* selective media, mannitol salt agar and chromID agar, were used for bacterial growing. Quantifying the *S. aureus* load varied significantly between the different sampling methods on naïve skin lesions ranging from mean 51 to 1.5 × 10^4^ CFU/cm^2^ (*p* < 0.001). The qualitative detection on naïve skin was highest with the two detergent-based techniques (86% each), while for tape stripping, this value was 67% (all on chromID agar). In comparison, mannitol salt agar was less sensitive (*p* < 0.001). The disinfection of the skin lesions led to a significant reduction of the *S. aureus* load (*p* < 0.05) but no complete eradication in the case of previously positive swab. The obtained data highlight the importance of the selected sampling method and consecutive *S. aureus* selection agar plates to implement further clinical studies for the effectiveness of topical anti-staphylococcal antibiotics. Other disinfection regimes should be considered in atopic dermatitis patients when complete de-colonisation of certain skin areas is required, e.g. for surgical procedures.

**Supplementary Information:**

The online version contains supplementary material available at 10.1007/s10096-021-04365-5.

## Introduction

*Staphylococcus aureus* is one of the most common pathogens associated with serious infections in almost all parts of the body. It is a well-known highly virulent pathogen that can cause a variety of diseases in humans, for example skin infections which range from rather benign such as folliculitis to more serious infections like furunculosis with a high risk of spreading with blood which may lead to fatal consequences. Osteomyelitis, pneumonia or endocarditis caused by *S. aureus* can also be the source of bloodstream infections and a life-threatening sepsis. Apart from that, up to 50% of healthy individuals are temporarily and 10–20% persistently colonised with *S. aureus* on intact skin and mucosae in the nose, hands, axilla and perineum. Together with a number of cell-bound virulence factors, *S. aureus* may secrete 30 or more specific products such as exotoxins that interfere with the host defence. Due to these different exotoxins, *S. aureus* is causing a variety of different systemic toxin-mediated diseases, from serious like staphylococcal toxic shock syndrome up to the frequent and mainly self-resolving staphylococcal food poisoning [1]. Additionally, there is a growing body of evidence linking *S. aureus* colonisation and degree of severity of atopic dermatitis (AD) [2]. However, since the 1970s, there is evidence that patients with AD are more likely to be colonised with *S. aureus* ranging from 30% to nearly 100% [3–5]. Finally, a systematic literature search summarised and confirmed that patients with AD are significantly more often colonised with *S. aureus* than healthy controls (odds ratio 19.7; 95% confidence interval 10.8–35.8) [6]. The detection of *S. aureus* in all these summarised studies was predominantly performed with culture-based swab or scrub methods based on a 1965 published survey by Williamson and Kligman [7]. While recent studies have relied on culture-independent methods, like DNA sequencing methods [8–10], they are not able to quantitatively assess the absolute degree of colonisation of vital *S. aureus* on affected skin and their load reduction after antimicrobial treatment.

Although the commonly used non-invasive detection methods (detergent scrubbing (DS), moist swabbing (MS) and tape stripping (TS)) have been known for decades to detect and quantify the bacterial load from human skin [11], no gold standard for the sampling method of culture-based *S. aureus* detection on skin lesions from patients with AD exists so far. To establish the optimal non-invasive *S. aureus* sampling method, DS, MS and TS were compared in this prospective, single-centre study. Thereby, we set out to determine the most sensitive method for qualitative and quantitative detection of living *S. aureus* on skin lesions from patients with AD on naïve and disinfected skin. This is of high interest for future clinical studies of topical anti-staphylococcal agents to measure their antimicrobial activity as well as for clinical routine use.

In addition, the sensitivity of two different *S. aureus* selection agars for detecting *S. aureus* and the effectiveness of common skin disinfection to remove *S. aureus* from AD skin lesions were investigated.

## Materials and methods

The protocol had been approved by the Ethical Board of the Medical University of Vienna (No. 2155/2016) and performed in accordance with the Declaration of Helsinki (1964), Good Clinical Practice guidelines of the European Commission and the Good Scientific Practice guidelines of the Medical University of Vienna. The study recruitment was conducted from February 2017 to March 2018 at the Department of Dermatology of the Medical University of Vienna during routine outpatient visits. All patients included were between 18 and 70 years old, male or female with signed and dated informed consent obtained and diagnosed with localised atopic dermatitis according to the Hanifin & Rajka criteria [12] (e.g. flexural eczema in a more or less symmetrical distribution on arms) with two individual lesions each covering an area between 40 and 300 cm^2^. The Eczema Area and Severity Index (EASI) score [13] has been routinely used at our clinic during the study period. Excluded were patients with a history of irritation following contact with the topical products Triton X-100, Octenidindihydrochlorid and/or 2-phenoxyethanol and/or systemic or topical treatment with antibiotics and/or use of antiseptic soaps at the tested skin lesion within 7 days before this study. For comparing three non-invasive sampling methods for detecting and quantifying *S. aureus* on skin lesions from AD, the sample size was calculated with at least 11 eligible *S. aureus*–positive patients for analysis of variance (based on www.statstodo.com). Because the *S. aureus* status was unknown at the beginning and AD lesions are colonised ranging from 30% to nearly 100% with *S. aureus*, we estimated a number of 30 AD patients with unknown *S. aureus* status to be sufficient for the purposes of this study.

### Skin disinfection

Two localised AD skin lesions, e.g. flexural eczema in a more or less symmetrical distribution on arms of an area between 40 and 300 cm^2^, were randomly selected, one for disinfection while the other skin lesion remained naïve. First, one randomly selected skin lesion was disinfected with a one-time wipe disinfection with the antiseptic agent Octenisept® containing 0.1 g Octenidindihydrochlorid and 2 g 2-phenoxyethanol per 100 ml. After an impact time of at least 1 min, the three sampling methods were performed on a defined skin area (i.e. 4.8 cm^2^ for DS, 4.5 cm^2^ for MS and 3.8 cm^2^ for TS) on the disinfected and also on the naïve skin lesion.

### Performing the non-invasive bacterial sampling methods

At the study visit, three non-invasive sampling methods were compared concomitantly. The techniques were always being performed in the same order for all subjects and both skin lesions.

#### Detergent scrub technique

A cylinder with a defined internal area of 4.8 cm^2^ was held firmly against the affected area and filled with 2.5 ml of sterile buffered non-ionic wash fluid (0.1% Triton X-100 in 0.075 M sodium phosphate buffer, pH 7.9) as initially described by Williamson and Kligman in 1965 [7]. The affected skin surface within the ring was rubbed firmly with a hard stick (Teflon policeman) for 1 min. After this, all the wash fluid was aspirated by a pipette from the inner cylinder and transferred to sterile tubes.

#### Moistened swab technique

A circle template with a defined internal area of 4.5 cm^2^ was applied to the affected area and the limited skin area swapped with a sterile cotton swap moistened with sterile buffered non-ionic wash fluid (0.1% Triton X-100 in 0.075 M phosphate buffer, pH 7.9) 3 times; the swap was then submerged in 1 ml wash fluid [14]. The swap buffer solution was vortexed mildly to release bacteria*.*

#### Tape stripping technique

A tape strip (D-SQUAME Standard Sampling Discs, CuDerm, USA) of a defined size of 3.8 cm^2^ was applied with gentle pressure to the lesional skin surface for 1 min by using a sterile gloved hand [15, 16]. After peeled off from the skin, the tape strip was transferred to a tube in a sterile way and submerged in wash fluid (0.1% Triton X-100 in 0.075 M phosphate buffer, pH 7.9). The tape strip containing wash fluid was vortexed gently.

### Microbiological processing

After performing the three methods, all collected liquid samples were immediately processed (but not later than 4 h after collecting) at the microbiology laboratory. To detect the bacterial load (colony-forming units (CFU)/ml) at each method, a serial dilution method was performed according to the Clinical and Laboratory Standards Institute (appendix 27). After incubation at 37 °C over 24 h, the *S. aureus*–specific colonies were counted. *S. aureus*–specific colonies were defined according to the manufacturer’s instructions at mannitol salt agar (MSA; Becton Dickinson GmbH, Germany) as medium-sized yellow colonies with yellow surrounding medium and at *S. aureus* chromID (SAID; bioMérieux, France) green colonies. MSA were used for samples from all patients (*n* = 30) and SAID selection agar for samples from the 10^th^ patient on continually (*n* = 21). The amount of bacterial load was indicated in CFU per cm^2^ of the investigated AD skin area. All *S. aureus* strains were routinely confirmed on species level by matrix-assisted laser desorption/ionisation time-of-flight mass spectrometry (MALDI-TOF, Bruker Daltonics, Bremen, Germany).

### Statistics

The statistical significance (*p* < 0.05) of the measured difference of the quantitative detection of *S. aureus* with the three different sampling methods was determined by the Friedman test; furthermore, the difference on naïve versus disinfected AD skin and on the two different *S. aureus* selection agar plates was determined by the Wilcoxon test. The statistical significance (*p* < 0.05) of the different qualitative *S. aureus* detection (yes/no) by two *S. aureus* selective media was estimated by the McNemar test.

## Results

In a total number of 30 patients, each suffering from AD, the three different non-invasive sampling methods (DS, MS, TS) were successfully performed on a disinfected and symmetrically distributed naïve AD skin lesion. The included patients with AD display a representative sample of a larger population of AD patients with mainly moderate disease according to the EASI score. Patient characteristics and disease severity are summarised in Table 1.

**Table 1:** Patient characteristics

Patients, *n* (female)	30 (16)
Age, years; median (minimum–maximum)	31.1 (19–69)
EASI^a^ score, *n* (%)	
0.1–1.0 (almost clear) 1.1–7.0 (mild) 7.1–21.0 (moderate) 21.1–50.0 (severe) 50.1–72.0 (very severe)	0 3 (10%) 22 (73%) 5 (17%) 0

^a^Eczema Area and Severity Index score

**Fig. 1:**
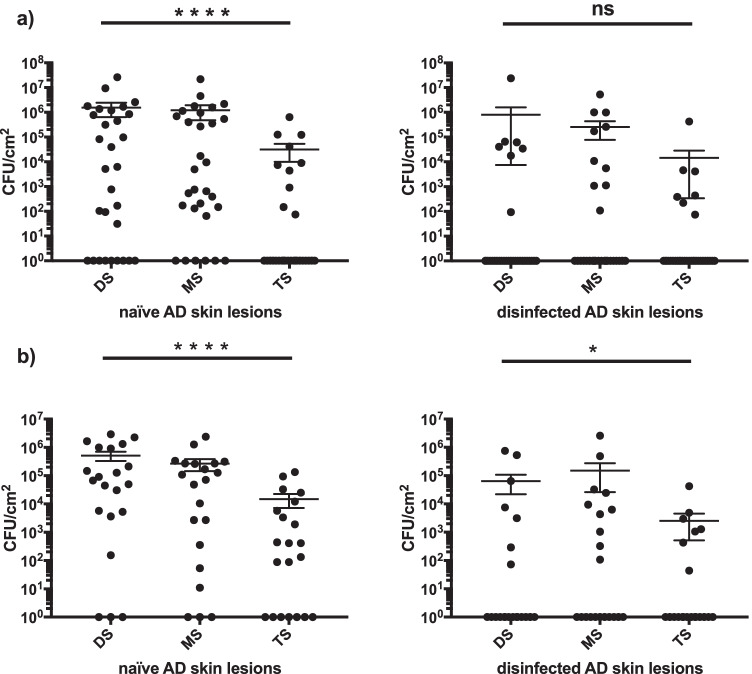
Quantitative detection of *S. aureus* by using different detection methods and selective media (mean and standard error of the mean values). **a** Quantitative detection of *S. aureus* by using selective media MSA (*n* = 30). **b** Quantitative detection of *S. aureus* by using selective media SAID (*n* = 21). **Abbreviations**: ****p < 0.0001; *p < 0.05; ns = *p* > 0.05; MSA, mannitol salt agar; CFU, colony-forming units of *Staphylococcus* *aureus*; SAID, *Staphylococcus aureus* chromID; DS, detergent scrubbing; MS, moist swabbing; TS, tape stripping; AD, atopic dermatitis

The sampling methods differed significantly in quantitative detection of *S. aureus* on naïve skin lesions (*p* < 0.001). The TS technique was notably less effective on naïve skin lesions compared to detergent-based methods like MS and DS, with the latter two showing no significant difference. After standardised disinfection, colonisation with *S. aureus* was significantly reduced (*p* < 0.05) independent of the detection method and selective media. Nevertheless, *S. aureus* was not fully eradicated and 47.6% were still colonised with *S. aureus*, as detected with MS, the most sensitive method. The results of the quantitative detection of *S. aureus* are summarised in Table 2 and Fig. 1.

**Table 2:** Quantitative detection of *S. aureus* by three different sampling methods collected on naïve and disinfected atopic dermatitis skin lesions and cultivated on two different *S. aureus* selective media

*S. aureus* selective media	Sampling methods	CFU^a^ × 10^4^/cm^2^ Mean (SD)		CFU^a^ × 10^4^/cm^2^ Median (percentile 25; 75)	
		Naïve skin	Disinfected skin	Naïve skin	Disinfected skin
MSA (*n* = 30)	DS	155.4 (495.2)	80.6 (437.3)	0.5 (0; 92.2)	0 (0; 0.002)
	MS	120.3 (398)	25.8 (98.6)	0.2 (0.01; 75.5)	0 (0; 0.2)
	TS	3.2 (11.8)	1.5 (7.8)	0 (0; 0.2)	0 (0; 0.002)
SAID (*n* = 21)	DS	51.9 (85.2)	6.5 (19.5)	6.8 (0.5; 95.8)	0 (0; 0.2)
	MS	26.9 (55.9)	15.1 (57.1)	7.1 (0.02; 27)	0 (0; 0.8)
	TS	1.5 (3.5)	0.3 (0.9)	0.04 (0; 0.9)	0 (0; 0.08)

^a^Colony-forming units of *S. aureus*

**Fig. 2:**
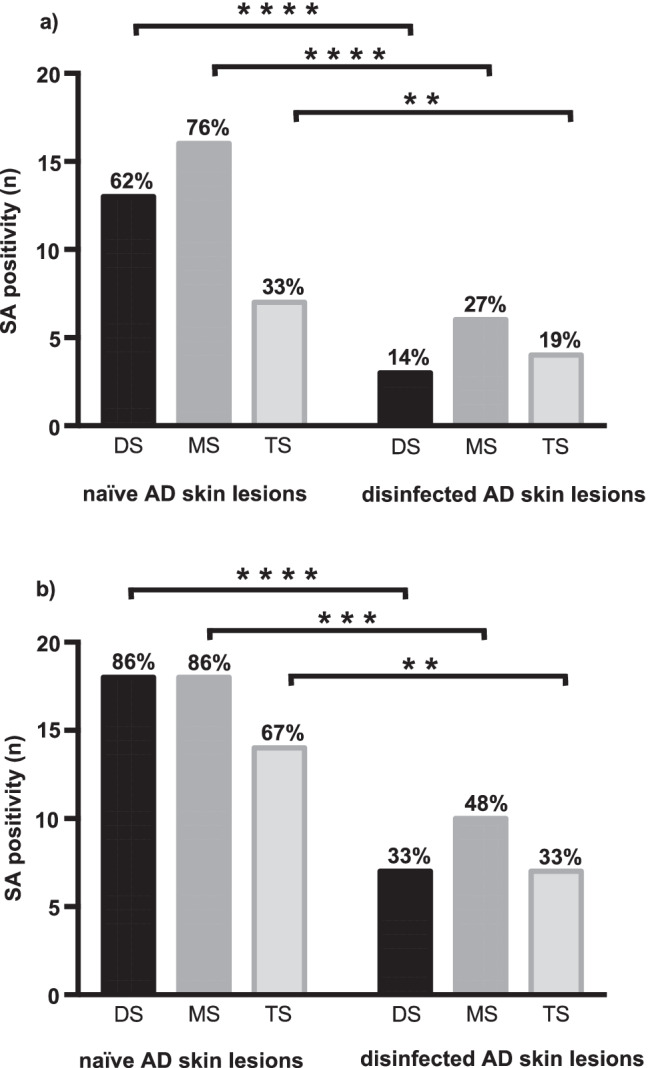
Qualitative detection of *S. aureus* by using different detection methods and selective media. **a** Qualitative detection of *S. aureus* by using selective media MSA (*n* = 21). **b** Qualitative detection of *S. aureus* by using selective media SAID (*n* = 21). **Abbreviations**: ****p < 0.0001; ***p < 0.001; **p < 0.1; SA, *S. aureus*; MSA, mannitol salt agar; SAID, *S. aureus* chromID; DS, detergent scrubbing; MS, moist swabbing; TS, tape stripping; AD, atopic dermatitis

The most sensitive sampling method for *S. aureus* detection on naïve and disinfected AD skin lesions was the MS technique in combination with chromogenic SAID selective media where the detected rate of colonisation was 85.7% on naïve skin lesions respectively and 47.6% on disinfected skin lesions compared with the lowest rate (33.3% respectively 19.1%) by using the TS technique in combination with MSA. The SAID selective media was more sensitive for the quantitative detection of *S. aureus* compared to MSA (*p* < 0.001). All results of qualitative detection of *S. aureus* are summarised in Tables 3 and 4 and in Fig. 2. The direct comparison of bacterial load (CFU) detected on MSA and SAID plates from samples collected with the three different sampling methods from naïve AD skin lesions showed on both agar plates a significant difference (supplementary Fig. 1). On disinfected AD skin lesions, the difference in bacterial density (CFU) between the collection techniques was significant only on SAID in direct comparison (supplementary Fig. 2).

**Table 3:** Comparison of two different *S. aureus* selective media with cultivated samples of three different sampling methods collected on naïve and disinfected skin lesions

		*S. aureus* positive					
		MSA (*n* = 21)		SAID (*n* = 21)		∆^a^ SAID − MSA	
Atopic dermatitis skin lesions	Sampling methods	*n*	%	*n*	%	*n*	%
Naïve	DS	13	61.9	18	85.7	5	+ 23.8
	MS	16	76.2	18	85.7	2	+ 9.5
	TS	7	33.3	14	66.7	7	+ 33.4
Disinfected	DS	3	14.3	7	33.3	4	+ 19.0
	MS	6	28.6	10	47.6	4	+ 19.0
	TS	4	19.1	7	33.3	3	+ 14.2
∆^a^ naïve − disinfected	DS	10	− 47.6	11	− 52.4		
	MS	10	− 47.6	8	− 38.1		
	TS	3	− 14.1	7	− 33.4		

^a^Difference

**Table 4:** Direct comparison of the qualitative detection of *S. aureus* from non-invasive skin bacteria sampling by two different *S. aureus* selective media

		Mannitol salt agar		Total
		*S. aureus* positive	*S. aureus* negative	
chromID agar	*S. aureus *positive	40	34	74
	*S. aureus* negative	9	43	52
Total		49	77	126

## Discussion

Atopic dermatitis is a frequent skin disease with an observed association of increased *S. aureus* colonisation [6]. Nevertheless, there is currently no defined gold standard for the sampling for culture to detect living *S. aureus* on skin lesions from AD patients and other forms of chronic skin conditions. Therefore, the three most promising non-invasive *S. aureus* sampling methods, two detergent-based (DS, MS) and one tape stripping (TS) methods, were compared in this study [11]. At first, the culture-based qualitative detection of *S. aureus* and the change of the viable *S. aureus* load after disinfection on AD skin lesions of patients with predominantly moderate disease according to the EASI score were investigated by these three different sampling methods. Results for these two main endpoints clearly differed between the sampling methods used and show the importance of the selected method in the clinical setting and research use before and after disinfection or antimicrobial treatment.

In detail, the highest sensitivity to detect *S. aureus* culture based on AD skin lesions and the persistence of *S. aureus* after disinfection was found with the detergent-based methods (DS or MS) in combination with the SAID selection agar. The sensitivity results differed clearly on naïve AD skin lesions, from 86% (used DS or MS method with SAID plates) to 33.3% (used TS method with MSA plates). Therefore, the prevalence of *S. aureus* colonisation was up to 86% on investigated naïve AD skin lesions where the density of bacteria on the skin lesions is naturally the highest, while the pooled prevalence of *S. aureus* colonisation reported in a systematic review of 81 studies was 70% (95% confidence interval 0.66–74). However, in a subgroup analysis from severe AD, the prevalence was comparable to our results [6] although our patients were mainly suffering from moderate AD (73% according to EASI score). *S. aureus* could be detected in all AD severity levels. The used EASI score, along with the two scores SCORAD and POEM, is one of the best-validated outcome measures for atopic dermatitis [17] and over the last years well established at our clinic to estimate the severity of atopic dermatitis. The data collected shows how different the culture results can be depending on the sampling method. Naïve skin lesions were clearly defined without topical or systemic antibiotics or local antimicrobial active soaps, nor was any sampling performed under cortisone or dermatological local therapy in the course of a (usually initial) evaluation of an episode of atopic dermatitis. The eradication rate of *S. aureus* after the used disinfection procedure reduced the bacterial load significantly independent from the used sampling method or *S. aureus* selection agar plates but surprisingly was not fully effective on AD skin lesions against *S. aureus* in a major proportion of patients. An octenidine-based antiseptic agent was chosen because it is approved for use on skin and mucous membranes and has good bactericidal activity against *S. aureus* (comparable to chlorhexidine) and good tolerability also on sensitive skin [18]. According to the used sampling method and *S. aureus* selection agar, the persistence rates were 48% (38% difference compared to non-disinfected skin) using the MS method and SAID selective media to 19% (14% difference compared to non-disinfected skin) using the TS method and MSA at disinfected AD skin lesions (Table 3). The antiseptic agent was applied only once for an impact time of at least 1 min, which may have been too short for effective decolonisation or should be repeated several times. However, the difference in the detection of living *S. aureus* before and after therapy is important, for example, in clinical trials to demonstrate the efficacy of new antiseptic agents to reduce the *S. aureus* load.

The detergent-based methods were significantly better to detect *S. aureus* on AD skin lesions. The buffered non-ionic detergent (0.1% Triton X-100 in 0.075 M sodium phosphate buffer, pH 7.9) first described by Williamson and Kligman [7] used for the scrubbing and swabbing method as sampling fluid was well tolerated without any side effects or skin irritations.

Remarkable are the weak results of the TS method to detect viable *S. aureus* on pathological AD skin lesions with high bacterial load. This is in contrast to previous results for skin microbiome by next-generation sequencing and culture-based studies, where even the culture-based study showed a greater number and wider variety of viable skin bacteria by the TS than the swabbing method [19]. However, compared to our study, the sample size was small (*n* = 7 versus 30), the skin was always healthy, the detergent buffer differed and *Staphylococcus epidermidis* was the lead detected bacterium; *S. aureus* was only detected once.

Sellotape stripping is in general an interesting method and easy to perform [19]. Only one tape strip per sample was used as is common in daily practice; this can be seen as a limitation of the study results. Another limitation could be that not all bacteria are successfully transferred from the foil by submerged in the washing fluid and vortexed gently. However, this is generally a disadvantage of the method, because in case of transfer of the tape strip directly on the agar plate also not all bacteria are successfully transferred to the agar medium or by leaving the tape strip in situ on the agar medium, the oxygen supply for the growth of aerobic and facultatively anaerobic bacteria such as *S. aureus* is inhibited and this could also falsify the results for the detection of growing *S.* *aureus*. A known disadvantage of the tape stripping technique is certainly that only bacteria on the skin surface are collected and the bacteria in the depth of the skin structure are not captured [16]. We suspect because 25% of the cutaneous bacteria population is localised in the depths of the skin structure and hair follicles [20] and *S. aureus* is found in the upper and the lower layers of the epidermis [21] and hair follicles [22] which may be a reason for the poor results with this particular pathogen of this method.

As expected, the DS method was highly effective to collect quantitative *S. aureus* from skin as has already been demonstrated by its widespread use for research purposes [11]. However, it was the most complex sampling method studied in our series. In addition, it was slightly unpleasant for the patient due to the necessary firm rubbing with a Teflon policeman for 1 min at the sensitive AD skin lesion as compared to the others. Although for detection of superficial skin bacteria this method is still considered as gold standard for the specific qualitative and quantitative detection of *S. aureus* on AD skin lesions, it was only equally effective to the less complex and less unpleasant MS method. Different kinds of swabs are often used routinely as a sampling method on intact or damaged skin. Generally, swab-based sampling methods for skin bacteria have a poor reputation among researchers mainly because of improper use of dry swabs [11]. For standardised MS performed with a non-ionic detergent on a defined skin area for detection of *S. aureus* on lesional AD skin, our study nevertheless showed excellent results. In addition, the handling of swabs is easy and quick and the defined skin area is simply marked with a sterile ring as a template. Former studies showed that swab-based methods collect mainly bacteria from the superficial skin layer and scrub-based methods collect the superficial skin cells and the associated microbes [8]. Indeed, we cannot rule out that for other settings a difference in detecting *S. aureus* in AD skin lesions might exist between the MS and DS methods.

In our study, we could also demonstrate that the choice of the *S. aureus* selection agar is an important issue. Overall, MSA was not without difficulties in identifying *S. aureus* due to difficulties in differentiating between *S. aureus* and coagulase-negative staphylococci why *S. aureus* strains were always confirmed by MALDI-TOF. The growing *S. aureus* colony resulted in yellow colouration of the agar in an area bigger than the diameter of the single colony, which resulted in a partly insufficient discrimination when the bacterial load was high and the single colonies were too close together despite using the highest dilution level. Therefore, after the first 9 patients, the SAID selective media were used, a chromogenic agar medium known to be highly sensitive and specific for detecting staphylococci and specifically identifying *S. aureus* [23]. The two different *S. aureus* selection agar plates showed significant differences by detecting *S. aureus*. Interestingly, MSA was significantly less sensitive regarding qualitative detection of living *S. aureus* compared to SAID agar as shown in detail in Table 4. Similar results were previously described with *S. aureus* chromogenic agars using clinical samples, i.e. from nasal swab specimens [24, 25] and food [26]. The importance of the choice of the selection agar was also shown by a subanalysis in direct comparison of the same sample on both plates, where on low bacterial load (on disinfected AD skin lesions) a significant difference between the sampling methods could be shown only with SAID plates (see supplementary Figs. 1 and 2).

## Conclusion

The obtained data highlight the importance of the selected sampling method and consecutive *S. aureus* selection agar plates. To implement further clinical studies for the effectiveness of topical anti-staphylococcal antibiotics and to study the links between *S. aureus* colonisation and AD, it is essential to standardise the used sampling technique during the whole study and for all investigators. The obtained data in our study clearly suggest the use of detergent-based methods like scrubbing or swabbing. A chromogenic *S. aureus* selection agar additionally improves the results. Due to the convenient handling and less sampling associated burden for the AD patient, the standardised moistened swab technique on a defined skin area should be considered the standard sampling method for *S. aureus*.

## Supplementary Information

Below is the link to the electronic supplementary material.

Supplementary file1 (DOCX 169 KB)

## Data Availability

The data generated and analysed in this study are available from the authors on request.
