# Supplementary material for: Comparison of non-invasive Staphylococcus aureus sampling methods on lesional skin in patients with atopic dermatitis
Source: Eur J Clin Microbiol Infect Dis. 2021 Nov 4;41(2):245–52. doi: 10.1007/s10096-021-04365-5 (PMC8770445; doi:10.1007/s10096-021-04365-5)
Supplement: Supplementary file 1 — Supplementary file1 (DOCX 169 KB) [file 10096_2021_4365_MOESM1_ESM.docx]

**Supplementary figure 1**:

**Comparison of sampling methods and agar plates on naïve skin of individual patients.**

**Abbreviations for supplementary figures 1 and 2:**

MSA, mannitol salt agar; SAID, *S. aureus* chromID; CFU, colony forming units of *S. aureus*; DS, detergent scrubbing; MS, moist swabbing; TS, tape stripping.

**Supplementary figure 2**:

Comparison of sampling methods and agar plates on disinfected skin of individual patients.

 **Legend for supplementary figures 1 and 2:**

For comparison of individual patients using different sampling methods on MSA plates (A) and SAID plates (B) a Friedmann test was used. For comparison of different plates using the DS method (C), MS method (D) or TS methods (E) a Wilcoxon matched-pairs signed rank test (two tailed) was used (**** p < 0.0001; *** p < 0.001; ** p < 0.01; * p < 0.05; ns = p > 0.05).
